# A longitudinal analysis of serum adiponectin levels and bone mineral density in postmenopausal women in Taiwan

**DOI:** 10.1038/s41598-022-12273-7

**Published:** 2022-05-16

**Authors:** Tong-Yuan Tai, Chi-Ling Chen, Keh-Song Tsai, Shih-Te Tu, Jin-Shang Wu, Wei-Shiung Yang

**Affiliations:** 1 Department of Internal Medicine, National Taiwan University Hospital, Taipei, Taiwan; 2 Graduate Institute of Clinical Medicine, College of Medicine, National Taiwan University, 7 Chung-Shan South Road, Taipei, 100 Taiwan; 3 Graduate Institute of Epidemiology and Preventive Medicine, College of Public Health, National Taiwan University, Taipei, Taiwan; 4 Department of Surgery, National Taiwan University Hospital, Taipei, Taiwan; 5 Department of Laboratory Medicine, National Taiwan University Hospital, Taipei, Taiwan; 6 Division of Endocrinology and MetabolismDepartment of Internal Medicine, Changhua Christian Hospital, Changhua, Taiwan; 7 Department of Family Medicine, National Cheng Kung University, Tainan, Taiwan; 8 Hepatitis Research Center, National Taiwan University Hospital, Taipei, Taiwan

**Keywords:** Biomarkers, Endocrinology, Medical research

## Abstract

Since bone and fat mass are derived from mesenchyme in early development, adipokines secreted by adipose tissue may have an effect on bone metabolism. The relationship between adiponectin and bone mineral density (BMD) has been inconsistent in previous reports, with results being dependent on age, gender, menopausal status and bone sites. We investigated the relationship between serum adiponectin levels and the BMD of proximal femur and vertebrae bones in a 96-week longitudinal study of post-menopausal women with repeated measures of both. Linear regression models were used to determine the relation between adiponectin and BMD at each time point cross-sectionally, and a generalized estimating equation (GEE) model was used to investigate the longitudinal trends. Among 431 subjects, 376 (87%) provided baseline adiponectin measurements and 373 provided more than two measurements for longitudinal analysis. The means of serum adiponectin and BMD decreased with time. In linear regression models, adiponectin at baseline, the 48th week and the 96th week appeared to be inversely associated with BMD of proximal femur bone, but not lumbar spine after adjusting for age and various confounders. However, they all turn insignificant with further adjustment of body mass index. The inverse association between adiponectin and BMD of proximal femur is substantiated by all generalized equation models. Before adding the BMI in the model, the increase of 1 mg/dL of adiponectin can accelerate the decrease of proximal femur BMD by 0.001 (SE = 0.0004, p = 0.008). With BMI in the model, the drop rate was 0.0008 (SE = 0.0004, p = 0.026) and remained similar with further adjustment of two bone turnover markers. In this longitudinal analysis with both adiponectin and BMD measured at three time points, we demonstrate that with the increase of adiponectin level, the decline of proximal femur BMD in postmenopausal women accelerated during a period of 96 weeks.

## Introduction

Bone has a very tight link with fat in embryonic development. Both osteoblasts and adipocytes originate developmentally from mesenchymal stem cells^1^. As a result, there have been significant interest and controversies surrounding the relationship of fat mass to bone mass, and even to fracture risk^2–4^. Moreover, the function of adipose tissue nowadays is no longer confined only to lipid depots. In the past decades, adipose tissue has been well demonstrated to play critical roles similar to an endocrine organ^5^. The humoral factors secreted by adipose tissues were shown to have profound impact on our physiological homeostasis. These factors secreted by the adipose tissue were given the names adipokines or adipocytokines. Among all adipokines, the blood level of adiponectin appears to be most relevant to bone mineral density (BMD) irrespective of menopausal status in women^6,7^.

Adiponectin and adiponectin receptors were then demonstrated to be present in primary human osteoblasts from femur and tibia^8^. Lenchik and colleagues were the first to report a significant negative association of serum adiponectin levels with both areal and volumetric BMD in adults, even after adjusting for whole body fat mass in men and women^9^. Soon afterwards, an inverse relationship between plasma adiponectin and BMD in non-diabetic female adolescents was reported^10^. However, this relationship turned insignificant after adjusting body mass index (BMI) or fat mass. To date, the roles of adiponectin in terms of bone metabolism remain incongruent in basic research^7^. On the other hand, the negative correlation between circulating adiponectin level and BMD of various skeletal sites appears consistent in human studies^6,11^. However, the majority of earlier human observations are cross-sectional^6,12^. Several longitudinal observational studies with repeated BMD measurements focusing on post-menopausal women were all based on only baseline adiponectin levels with inconsistent results^13–16^. The protective effect of obesity on bone health has been termed the “obesity paradox”^17^. Both the weight-bearing factor (physical weight) and non-weight-bearing factors (such as adiponectin) were proposed to affect bone health^5^. Delineation of the relationship between adiponectin and BMD may provide further insight about the biology of the obesity paradox. Previously we conducted a 2-year randomized placebo-controlled double-blind clinical trial to observe the effect of soy isoflavone, a phytoestrogen on BMD in 431 Taiwanese post-menopausal women^18,19^. Since all participants had serum adiponectin levels measured at baseline, 48th week and 96th week, this cohort also provides us with a unique opportunity to examine the relationship between adiponectin and BMD longitudinally. We aim to determine whether the change of adiponectin could accelerate or slow down the decrease of BMD in postmenopausal women.

## Methods

### Study population

As reported previously, the study cohort is from a randomized placebo-controlled double-blind clinical trial^18^. 431 Taiwanese postmenopausal women between the age of 45 and 65 were included. All the subjects were recruited between December 2004 and January 2006. The study was approved by human research ethics committees of the three participating hospitals (NTUH, CCH and NCKUH) and the National Health Research Institutes of Taiwan, and written informed consent was obtained from each individual. The study protocol was in accordance with the Declaration of Helsinki and Good Clinical Practices Guidelines.

The cessation of menses was at least 12 months, but less than 10 years. BMI was 18.5–30 kg/m^2^. BMD of the second to fourth lumbar vertebrae was 1 SD below the young adult female mean value (T-score <  − 1). To confirm menopausal status, serum follicle stimulating hormone (FSH) concentration had to be > 40 IU/L and estradiol concentration (E2) < 40 pg/mL. The exclusion criteria were quite extensive as described in said previous report^18^. In short, the exclusion criteria include history of fractures or metabolic bone diseases; cancer history; undiagnosed vaginal bleeding or endometrial hyperplasia; cardiovascular disorders; diabetes with HbA1c ≥ 10%; blood pressure ≥ 180/100 mmHg; hypothyroidism; alanine aminotransferase (ALT) > twofold upper limits, serum creatinine > 2 mg/dL; use of hormone replacement therapy, estrogen receptor modulators, or phytoestrogen within the previous 3 months; use of fluoride, calcitonin, chronic systemic corticosteroid, or any other treatment affecting BMD within the previous 6 months; or use of bisphosphonate within the previous 1 year, or an accumulative usage of bisphosphonate for more than 3 months more than 1 year ago. For those who had undergone hysterectomy, their ages had to be between 50 and 60, with FSH and E2 concentrations as previously stated. The allocation of the participants and drug products in the clinical trial were as described in said previous report^18^.

### Biochemical and other covariate measurements

After overnight fasting, venous blood was collected for biochemistry and biomarker measurements at baseline, 48th and 96th weeks. The biochemical measurements were largely routine clinical laboratory tests, including fasting plasma glucose, lipid profile, liver and renal function tests, and high sensitivity C-reactive protein^18^. Bone-specific serum alkaline phosphatase (BAP, Beckman Access Ostase, Fullerton, CA, USA) and N-telopeptide of type 1 collagen (NTx, Vitros Immunodiagnostic Products, Ortho-Clinical Diagnostics, Buckinghamshire, UK) were examined at baseline and 48 and 96 weeks as previously reported^18^. Adiponectin level was measured by a commercial immunoassay kit following manufacturer’s protocol (Asone international Inc., Santa Clara, CA) as previously reported^20^. Before the assay, the 10 μl sample was diluted for 10 × with dilution buffer, subjected to boiling for 5 min, then diluted 5100-fold. The International Physical Activity Questionnaire-Short Form, 24-h diet recall, and the Isoflavone Basic Diet Information Food Frequency Questionnaire were used to measure physical activity levels (total METs per week), daily energy and calcium intake at baseline, 48 and 96 weeks^18^. Participants were asked to maintain their habitual diet and exercise patterns, and the same dietitians documented this using validated questionnaires with personal interviews.

### Bone mineral density assessment

BMD of lumbar spine (L2–L4) and right total proximal femur was measured by dual-energy X-ray absorptiometry (DXA) at baseline and 24, 48, 72, and 96 weeks after randomization. The DXA equipment used and some details of measurement were as previously reported^18^.

### Statistical analysis

Continuous data were presented as means with standard deviations, while the categorical data as numbers and percentages. The baseline serum adiponectin levels were categorized into quartiles (< 8.9, 8.9–< 12.57, 12.57–< 16.29, ≥ 16.29 mg/dL) and the comparison of selected baseline characteristics were conducted by ANOVA for continuous variables and Chi-square for categorical variables. Simple linear regression was used to evaluate the correlation between BMD and serum adiponectin levels at three time points. Generalized estimating equation (GEE) models were used to investigate the longitudinal trends of serum adiponectin levels on the change of BMD, after controlling for the effect of age, time, isoflavone treatment, hospital sites, history of diabetes, hypertension and hyperlipidemia, body mass index, average total METs spent and total calories consumed and bone turnover markers including bone-specific serum alkaline phosphatase and N-telopeptide of type 1 collagen. All statistical analyses were conducted using STATA (version 13) with p value < 0.05 as statistically significant.

## Results

Among the 431 postmenopausal women who participated in the clinical trial, 376 (87%) had serum adiponectin levels at baseline and formed the study cohort, and 373 had more than two measurements available for longitudinal analysis. We divided the subjects into quartile groups based on their baseline serum adiponectin levels (< 8.9, 8.9–< 12.57, 12.57–< 16.29, ≥ 16.29 mg/dL) with 94 women in each group to evaluate the relationship between serum adiponectin and selected baseline characteristics (Table 1). There was no difference in age and menopausal duration among the 4 groups. As expected, the subjects with higher serum adiponectin tended to have lower BMI, higher daily physical activity, and were less likely to have hyperlipidemia (Table 1). The women in the higher quartiles of serum adiponectin had lower right total proximal femur T-score at all three time points, while there was no significant difference in lumbar spine BMD, T-score and proximal total femur BMD (Table1).

**Table 1:** Selected characteristics and bone mineral density (BMD) by serum adiponectin quartiles.

Adiponectin at baseline	Q 1 (N = 94) (< 8.82) Mean (SD) /NO (%)	Q 2 (N = 94) (8.82–11.95) Mean (SD) /NO (%)	Q 3 (N = 94) (11.96–15.87) Mean (SD) /NO (%)	Q 4 (N = 94) (≥ 15.88) Mean (SD) /NO (%)	P-value
Age (yr)	55.4 (3.9)	55.0 (3.8)	55.3 (3.4)	55.0 (3.8)	0.816
BMI	23.5 (2.6)	22.8 (2.6)	22.5 (2.4)	22.0 (2.1)	0.0004
Menopausal duration (yr)	5.5 (2.7)	5.5 (2.6)	4.8 (2.4)	5.1 (2.6)	0.225
History of hysterectomy	12 (12.8%)	10 (10.6%)	9 (9.6%)	9 (9.6%)	0.88
History of diabetes	1 (1.1%)	0	0	2 (2.1%)	
History of hypertension	21 (22.3%)	13 (13.8%)	11 (11.7%)	12 (12.8%)	0.158
History of hyperlipidemia	54 (57.5%)	49 (52.1%)	42 (44.7%)	31 (33.0%)	0.005
Cigarette smoking	0	1 (1.1%)	0	0	
Habitual alcohol consumption	2 (2.1%)	3 (3.2%)	4 (5.3%)	4 (5.3%)	0.89
Isoflavon treatment	44 (46.8%)	47 (50.0%)	50 (53.2%)	48 (51.1%)	0.85
Daily physical activity (total METs/week)	3719 (1106)	4179 (1566)	4592 (2139)	4865 (1895)	< 0.001
Daily energy intake (Kcal)	1512 (352)	1577 (374)	1586 (347)	1519 (352)	0.475
Daily calcium intake (mg)	460 (212)	533 (221)	493 (181)	537 (238)	0.121
**Lumbar spine BMD (g/cm**^**2**^**)**					
Baseline	0.88 (0.08)	0.89 (0.08)	0.90 (0.09)	0.89 (0.10)	0.754
48th week	0.88 (0.08)	0.89 (0.08)	0.89 (0.09)	0.88 (0.10)	0.871
96th week	0.87 (0.08)	0.88 (0.08)	0.88 (0.09)	0.88 (0.10)	0.599
**Lumbar spine T-score**					
Baseline	− 1.89 (0.69)	− 1.94 (0.67)	− 1.97 (0.77)	− 2.06 (0.74)	0.415
48th week	− 1.93 (0.72)	− 1.93 (0.66)	− 2.03 (0.76)	− 2.13 (0.76)	0.195
96th week	− 2.04 (0.70)	− 2.02 (0.65)	− 2.07 (0.74)	− 2.17 (0.78)	0.523
**Total proximal femur BMD (g/cm**^**2**^**)**					
Baseline	0.80 (0.08)	0.78 (0.09)	0.79 (0.09)	0.77 (0.10)	0.233
48th week	0.79 (0.08)	0.78 (0.09)	0.79 (0.09)	0.77 (0.10)	0.398
96th week	0.79 (0.08)	0.77 (0.08)	0.78 (0.09)	0.76 (0.10)	0.312
**Total proximal femur T-score**					
Baseline	− 0.90 (0.70)	− 1.07 (0.76)	− 1.08 (0.88)	− 1.40 (0.77)	0.003
48th week	− 0.93 (0.67)	− 1.13 (0.70)	− 1.11 (0.83)	− 1.36 (0.76)	0.01
96th week	− 0.97 (0.71)	− 1.20 (0.74)	− 1.17 (0.84)	− 1.42 (0.78)	0.01
**Bone alkaline phosphatase (μg/L)**					
Baseline	16.68 (5.89)	16.24 (5.83)	15.48 (5.42)	16.04 (5.99)	0.549
48th week	14.94 (5.37)	14.24 (4.67)	14.47 (4.76)	14.82 (4.53)	0.747
96th week	14.94 (4.69)	14.59 (4.57)	14.72 (4.82)	15.07 (4.52)	0.901
**Urinary N-telopeptide of type 1 collagen/creatinitine (nM BCE/mM)**					
Baseline	69.19 (35.6)	60.26 (27.5)	64.39 (24.1)	66.25 (59.8)	0.468
48th week	64.90 (26.8)	61.20 (29.7)	63.60 (26.3)	70.02 (43.8)	0.297
96th week	60.13 (24.0)	59.57 (25.5)	61.49 (24.5)	60.30 (25.3)	0.960

The means (SD’s) of serum adiponectin and BMD levels at the three time points, as well as the linear regression coefficients (SE’s) of adiponectin and BMD at each time point are shown in Table 2. The mean serum adiponectin levels decreased during two-year follow-up periods from 13.03 (5.50) to 12.68 (5.07) and 12.27 (4.90). The mean BMD for both lumbar spine and total proximal femur also declined with time 0.89 (0.09), 0.88 (0.09), 0.88 (0.09) at baseline, the 46th week and the 96th week respectively in lumbar spine BMD and the respective numbers were 0.79 (0.09), 0.78 (0.09), 0.78 (0.09) for total right femur BMD. To further clarify the relationship between serum adiponectin and BMD, multiple linear regression analyses with the adjustment of age, isoflavone treatment, hospital sites, history of diabetes, hypertension and hyperlipidemia as well as average total METs spent and total calories consumed, and the addition of BMI were conducted (Table 2). For lumbar spine BMD and t-score, none of the associations at baseline, the 24th week and the 96th week were statistically significant, although the negative relationship was present. For proximal femur BMD and t-score, the negative association was statistically significant at baseline, whereas those at the 48th week were of borderline significance in Model 1 (Table 2). Once BMI was put into the model, none of the above remained significant (Model 2 in Table 2).

**Table 2:** The association between serum adiponectin levels and lumbar spine/total proximal femur BMD at three time points using simple linear regression models.

	Serum adiponectin level (mg/dL)	Lumbar spine				Total proximal femur			
		BMD(g/cm^2^)		T-score		BMD(g/cm^2^)		T-score	
	Mean (SD)	Mean (SD)		Mean (SD)		Mean (SD)		Mean (SD)	
Baseline	13.03 (5.50)	0.89 (0.09)		− 1.97 (0.72)		0.79 (0.09)		− 1.09 (0.78)	
NTUH	13.69 (4.57)	0.87 (0.09)		− 2.26 (0.66)		–		–	
CCH	14.66 (6.08)	0.93 (0.09)		− 1.95 (0.74)		0.79 (0.09)		− 1.31 (0.72)	
NCKUH	10.89 (5.10)	0.87 (0.07)		− 1.69 (0.64)		0.78 (0.09)		− 0.90 (0.79)	
48th week	12.68 (5.07)	0.88 (0.09)		− 2.01 (0.73)		0.78 (0.09)		− 1.13 (0.75)	
NTUH	13.25 (4.24)	0.86 (0.09)		− 2.28 (0.66)		–		–	
CCH	14.30 (5.41)	0.92 (0.09)		− 2.04 (0.73)		0.79 (0.08)		− 1.33 (0.68)	
NCKUH	10.58 (4.80)	0.87 (0.08)		− 1.71 (0.68)		0.77 (0.09)		− 0.93 (0.76)	
96th week	12.27 (4.90)	0.88 (0.09)		− 2.08 (0.72)		0.78 (0.09)		− 1.18 (0.77)	
NTUH	12.88 (4.31)	0.86 (0.10)		− 2.33 (0.69)		–		–	
CCH	13.87 (5.21)	0.91 (0.08)		− 2.13 (0.70)		0.78 (0.09)		− 1.41 (0.70)	
NCKUH	10.16 (4.40)	0.86 (0.08)		− 1.78 (0.67)		0.77 (0.09)		− 0.96 (0.78)	
		β (SE)	P	β (SE)	P	β (SE)	P	β (SE)	P
**Model I**									
Baseline		− 0.0005(0.0009)	0.555	− 0.005(0.007)	0.465	− 0.003(0.001)	0.003	− 0.027(0.009)	0.003
48th week		− 0.001 (0.001)	0.205	− 0.011(0.008)	0.158	− 0.002 (0.001)	0.055	− 0.018 (0.009)	0.055
96th week		− 0.001(0.001)	0.230	− 0.011(0.008)	0.181	− 0.002 (0.001)	0.086	− 0.018 (0.010)	0.073
**Model II**									
Baseline		0.00006 (0.0009)	0.948	− 0.0008 (0.007)	0.916	− 0.002 (0.001)	0.116	− 0.014 (0.009)	0.118
48th week		− 0.0005 (0.001)	0.577	− 0.006 (0.008)	0.473	− 0.0006 (0.001)	0.589	− 0.005 (0.009)	0.571
96th week		− 0.0006 (0.001)	0.555	− 0.006 (0.008)	0.461	− 0.0007 (0.001)	0.534	− 0.007 (0.01)	0.475

Model I: adjusted for age, isoflavone treatment, hospital sites, history of diabetes, hypertension and hyperlipidemia as well as average total METs spent and total calories consumed.

Model II: Model I plus body mass index.

**Figure 1:**
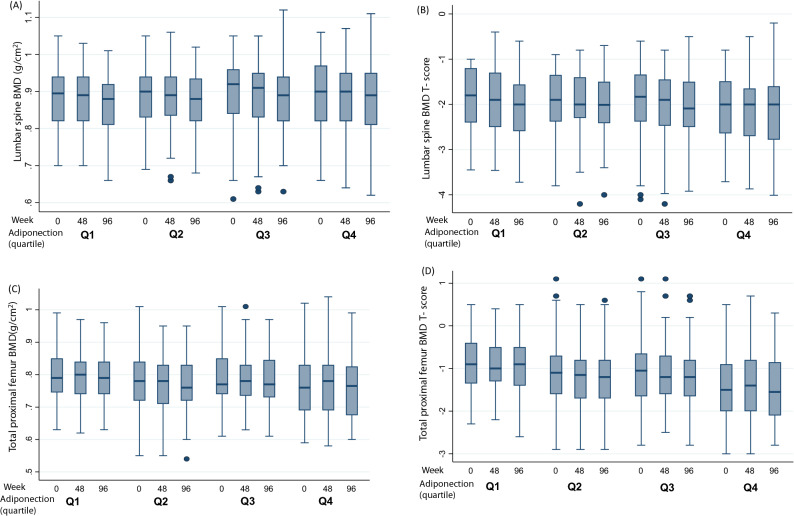
Whiskers plots of BMD and T-score at baseline, week 24 and week 96 in the quartiles of serum adiponectin levels: BMD (g/cm^2^) (**A**), T score (**B**) at lumbar spine and BMD (g/cm^2^) (**C**), T-score (**D**) at total proximal femur.

Since this is a longitudinal study, we then investigated the relationship between the changes in serum adiponectin levels and the changes in BMD overtime, by configuring the trends in Box plots (Fig. 1) and using GEE models to evaluate the association after the adjustment of other covariates (Table 3). The decrease of lumbar spine BMD and t-score with each visit (time trend) was shown in the first three quartiles of the baseline adiponectin levels, and most prominent among those in the third quartile (Fig. 1A,B). As to total proximal femur BMD and t-score, the decrease as adiponectin increase at each time point can be seen, while the trend within each quartile was different from lumbar spine BMD, in that the BMD levels increased at the 48th week, and dropped at the 96th week (Fig. 1C,D). Although the trend of BMD decreasing as adiponectin levels increased can be seen in the figure, regression models using repeated measurements of both adiponectin and BMD, and taking into consideration of other covariates, can provide more solid information. For lumbar spine BMD and t-score, the negative association between the changes in serum adiponectin levels and the changes in BMD after adjustment of age and time were significant (Model 1 and 2 Table 3). The remaining models with the adjustment of more variables for lumbar spine BMD levels were not significant (Table 3). In contrast, all the negative associations between the changes in serum adiponectin levels and the changes in proximal femur BMD and t-score were significant with various adjustments (Table 3). Before adding BMI to the model, an increase of 1 mg/dL of adiponectin could accelerate the decrease of proximal femur BMD by 0.001 (SE = 0.0004, p = 0.008) and t-score by 0.009 (SE = 0.003, p = 0.008). With BMI in the model, the drop rate was 0.0008 (SE = 0.0004, p = 0.026) for BMD and 0.007 (SE = 0.003, p = 0.025) for t-scores, respectively. The magnitude of decrease remained similar with further adjustment of two bone turnover markers (β = − 0.0008(0.0004), p = 0.029 for BMD, β = − 0.007(0.003), p = 0.028 for t-score, with the additional adjustment of serum BAP in model V of Table 3; β = − 0.0007(0.0004), p = 0.05 for BMD, β = − 0.006(0.003), p = 0.05 for t-score with the additional adjustment of urinary NTx/creatinine in model VI of Table 3.

**Table 3:** The association between the changes of serum adiponectin levels and changes of lumbar spine/total proximal femur BMD and t-score using generalized estimating equation (GEE) models.

Serum adiponectin level (mg/dL)	Lumbar spine				Total proximal femur			
	BMD(g/cm^2^)		T-score		BMD(g/cm^2^)		T-score	
	β (SE)	P	β (SE)	P	β (SE)	P	β (SE)	P
Model I	− 0.0004(0.0004)	0.264	− 0.007(0.003)	0.031	− 0.001 (0.0004)	0.013	− 0.011(0.003)	0.001
Model II	− 0.0004(0.0004)	0.243	− 0.007(0.003)	0.027	− 0.001 (0.0004)	0.011	− 0.001(0.003)	0.001
Model III	− 0.0006(0.0004)	0.121	− 0.005(0.003)	0.143	− 0.001 (0.0004)	0.008	− 0.009(0.003)	0.008
Model IV	− 0.0005(0.0004)	0.206	− 0.004(0.003)	0.239	− 0.0008(0.0004)	0.026	− 0.007(0.003)	0.025
Model V	− 0.0005(0.0004)	0.167	− 0.004(0.003)	0.195	− 0.0008(0.0004)	0.029	− 0.007(0.003)	0.028
Model VI	− 0.0003(0.0004)	0.373	− 0.003(0.003)	0.390	− 0.0007(0.0004)	0.050	− 0.006(0.003)	0.050

Model I: adjusted for time and time^2^.

Model II: adjusted for age, time and time^2^.

Model III: adjusted for age, time, time^2^, isoflavone treatment, hospital sites, history of diabetes, hypertension and hyperlipidemia as well as average total METs spent and total calories consumed.

Model IV: Model III plus body mass index.

Model V: Model IV plus bone alkaline phosphatase.

Model VI: Model IV plus urinary N-telopeptide of type 1 collagen/creatinitine.

## Discussion

In the past decades, adipose tissue has been considered an endocrine tissue affecting various physiological functions through endocrine or paracrine signaling^5^. Since bone and fat are developmentally related^21^, it is not surprising that adipocytes and their secretory adipokines may have significant effects on bone metabolism. Many have reported negative associations between blood adiponectin and BMD mainly in perimenopausal or postmenopausal women^22–24^. In contrast, this association has not always been observed in men and premenopausal women^25,26^. However, whether this association was confounded by fat mass parameters remains inconclusive^6^. In this longitudinal analysis in postmenopausal women with both serum adiponectin levels and BMD measured at three time points, we were able to demonstrate that with the increase of adiponectin level, the decline of BMD in the total proximal femur, but not in the lumbar spine, accelerated during a period of 96 weeks, with the adjustment of all other important covariates including BMI and bone turnover markers. Among 4 previous longitudinal studies that conducted separate analyses on postmenopausal women^13–16^, two reported inverse associations of adiponectin levels and the BMD of various sites cross-sectionally at baseline, but not the bone loss during follow-up^14,16^. One study found that the initial adiponectin level combining with specific body composition characteristics can predict loss of lumbar spine BMD over a 12-month period^13^. Another large study conducted in the U.S. with over 10 years of follow-up reported that adiponectin was associated with increased hip areal BMD loss, but not trabecular lumbar spine volumetric BMD loss^15^.

In our study, the negative relation between serum adiponectin and BMD was mainly present in the proximal femur bone, not in the lumbar spine. Since the total proximal femur BMD data from one of the participating medical centers (NTUH) were missing due to the software limitation of the DXA machine used, we performed sensitivity analyses to rule out the possibility that the association was totally, or in part, due to the missing data of one center. We limited the analysis to the two centers with complete data and found the results remained the same for lumbar spine BMD. We also repeated all analyses separately by the medical centers and the results were shown in supplement Tables 1 and 2. Similar to our results, some have reported this association primarily in the femur bone as well^27–29^. But others have also reported this relationship in both the femur bone and the vertebrate^16,30–34^, or in the lumbar spine alone^13^. Higher serum adiponectin levels were found to be associated with increased risk of osteoporotic facture of vertebrate, but not of long-bone in post-menopausal women^11^ or both in older men and women^35^. Therefore, the effects of adiponectin on BMD may not be bone site-specific based on the review of past literature^36^. Nevertheless, these studies were based on different BMD measurement tools with different bone sites, thus further study is still warranted.

The mechanisms underlying the negative association between adiponectin and BMD remain elusive. Interestingly, adiponectin knockout mice had reduced whole-skeleton mineral density^37^. Reduced bone mass in adiponectin-deficient mice may be caused by enhanced osteoclastogenesis and suppressed osteoblastogenesis^38^. In line with this, intracerebroventricular infusion of adiponectin increased osteogenic marker expression and trabecular bone mass in adiponectin knockout mice as well as in wild-type mice^39^. In contrast, it was also reported that adiponectin deficiency has no effect on BMD in mice, and in fact protected animals from ovariectomy-induced bone loss^40^. Taken together, adiponectin may not have a direct negative effect on BMD. Alternatively, increased bone marrow adipogenesis is a popular hypothesis to explain reduced BMD. Increased bone marrow adipogenesis is suggested to contribute to systemic hyperadiponectinemia and known to be associated with reduced BMD^41^. However, some have demonstrated that deficiency or over-abundance of bone marrow adipocytes did not significantly alter bone density^42,43^. Therefore, it is plausible to speculate the existence of a common upstream pathway leading to all three: bone marrow adipogenesis, increased adiponectin levels and reduced BMD. We propose that this common soil is highly likely to be the peroxisome proliferator-activated receptor γ (PPARγ). PPARγ is a master gene for adipogenesis. Activation of PPARγ in mesenchymal stem cells is involved in switching osteoblastogenesis into adipogenesis^1^. PPARγ-deficient mice with the PPARγ heterozygous genotype were shown to have higher bone mass with enhanced osteoblastogenesis^44^. In human studies, treatment with PPARγ agonist increased fat mass, elevated blood adiponectin levels and reduced BMD, and even increased fracture risk in females^45,46^. Therefore, endogenous activity of PPARγ signaling could be the mechanism responsible for the negative association between serum adiponectin levels and BMD.

Obesity and osteoporosis are both signs of ageing and contribute to age-dependent functional decline. However, obesity was shown to have protective effects on bone health, a phenomenon coined the “obesity paradox”^17^. This could be caused by weight-bearing effects, such as physical weight on bone remodeling. Alternatively, it could be related to non-weight-bearing factors, such as the negative effect of adiponectin demonstrated in this report. Beyond the scope of adiposity-related aspects addressed in this report, Ilesanmi-Oyelere et al. have demonstrated that lean mass rather than fat mass is associated with BMD of various sites^47^. This observation may highlight the importance of physical activity. Furthermore, we recently showed that adiposity and higher serum leptin level are associated with dynapenia (reduced muscle strength without sarcopenia)^48,49^. On the other hand, higher serum leptin reduced the risk of sarcopenia^49^. Taken together, the complex interplays among adipose tissue, skeletal muscle and bone revealed by these human studies warrant further investigation.

The main strength of this study is that we have measurements of both serum adiponectin levels and BMD at baseline, 48 weeks and 96 weeks; thus, we were able to analyze data using a robust regression model for repeated measurements that can take into consideration the time trend, main effect and other important covariates. With measurements of bone turnover markers at each time point, we were able to further adjust for their effects on the adiponectin-BMD association. In addition, the time intervals between each measurement were fixed at 48 weeks, which gave us a unique opportunity to observe the natural change of both serum adiponectin levels and BMD over time. Most of the previous studies in this literature have been cross-sectional, through which the time sequence of adiponectin affecting BMD or vice versa is hard to establish. To date, there has only been one longitudinal study^16^ with one set of follow-up data on BMD that had different time intervals for each individual and only baseline adiponectin levels. Because our study cohort was based on a randomized clinical trial, the participants were recruited based on strict inclusion and exclusion criteria to ensure that the population was representative of healthy postmenopausal women who were homogeneous in all aspects. Since the original trial supplement of isoflavone did not stop the decline of BMD, we can also observe the natural decline of BMD in this group of healthy postmenopausal women with mean menopausal period of 5 years (mean = 5.26, SD = 2.58).

We used three different models of instruments from different manufacturers to measure BMD, which may bring bias into our analysis. To guard against this possibility, several measures had been taken: (1) All BMD measurements were measured by the same certified technician using the same instrument in each medical center with good reliability and reproducibility^18^. (2) The raw BMD readings from three instruments were then standardized by taking the weights of 1.076, 1.0755 and 0.9522 for the lumbar spine at NTUH, CCH and NCKUH, respectively. As for the proximal femur BMD, the adjustment equations were as follows: BMD (By Norland in NTUH) × 1.012 + 0.026, BMD (By Hologic in CCH) × 1.008 + 0.006, and BMD (By Lunar in NCKUH) × 0.979–0.031. All the analysis was based on these adjusted BMD levels and BMD t-scores to ensure that the adiponectin-BMD association is not due to the difference in BMD measurements. (3) The measurement of serum adiponectin levels was conducted by a centralized laboratory. Nevertheless, the mean was significantly lower in NCKUH (Table 2), so we put medical centers into the regression model to neutralize this effect. (4) All the analyses were performed separately at each medical center, and the results were shown in supplement Tables 1 and 2. Basically, the results were similar, but the reduced power rendered most of them statistically insignificant.

In conclusion, we demonstrate that in this cohort of relatively young and healthy postmenopausal women with mean menopausal of 5 years, the increase of serum adiponectin levels would accelerate the loss of BMD at the proximal femur bone. Previous longitudinal studies with repeated BMD measurements were all focused on older women (> 70 years old). Thus, we provide further evidence that adiponectin levels start to influence bone health quite early in the menopausal timeline. When adiponectin starts to mediate BMD and the mechanisms behind it remains unclear and warrants further investigation.

## Supplementary Information

Supplementary Table 1.

Supplementary Table 2.
