# Supplementary material for: A longitudinal analysis of serum adiponectin levels and bone mineral density in postmenopausal women in Taiwan
Source: Sci Rep. 2022 May 16;12:8090. doi: 10.1038/s41598-022-12273-7 (PMC9110357; doi:10.1038/s41598-022-12273-7)
Supplement: Supplementary file 1 — Supplementary Table 1. [file 41598_2022_12273_MOESM1_ESM.docx]

**Supplement Table 1**: **The association between serum adiponectin levels and lumbar spine/total proximal femur BMD at three time points using simple linear regression models by different medical centers**

|  | **Serum adiponectin level**  **(mg/dL)** | **Lumbar spine** | | | | **Total proximal femur** | | | |
| --- | --- | --- | --- | --- | --- | --- | --- | --- | --- |
|  |  | **BMD** | | **T score** | | **BMD** | | **T score** | |
| **Model I** |  | **β (SE)** | **P** | **β (SE)** | **P** | **β (SE)** | **P** | **β (SE)** | **P** |
| **Baseline** |  |  |  |  |  |  |  |  |  |
| **NTUH** |  | 0.0009 (0.002) | 0.650 | 0.006 (0.014) | 0.654 | -- |  | -- |  |
| **CCH** |  | -0.0009 (0.001) | 0.535 | -0.007 (0.012) | 0.542 | -0.003 (0.001) | 0.068 | -0.02 (0.01) | 0.074 |
| **NCKUH** |  | -0.001 (0.001) | 0.407 | -0.011 (0.011) | 0.345 | -0.004 (0.002) | 0.018 | -0.033 (0.014) | 0.017 |
| **48^th^ week** |  |  |  |  |  |  |  |  |  |
| **NTUH** |  | -0.0007 (0.002) | 0.730 | -0.005 (0.015) | 0.727 | -- |  | -- |  |
| **CCH** |  | -0.001 (0.002) | 0.436 | -0.012 (0.013) | 0.377 | -0.002 (0.002) | 0.221 | -0.015 (0.012) | 0.217 |
| **NCKUH** |  | -0.002 (0.001) | 0.226 | -0.016 (0.013) | 0.215 | -0.003 (0.002) | 0.110 | -0.023 (0.014) | 0.115 |
| **96^th^ week** |  |  |  |  |  |  |  |  |  |
| **NTUH** |  | -0.0003 (0.002) | 0.883 | -0.002 (0.015) | 0.868 | -- |  | -- |  |
| **CCH** |  | -0.002 (0.002) | 0.221 | -0.017 (0.013) | 0.205 | -0.001 (0.002) | 0.366 | -0.013 (0.013) | 0.325 |
| **NCKUH** |  | -0.001 (0.002) | 0.359 | -0.013 (0.014) | 0.343 | -0.003 (0.002) | 0.129 | -0.025 (0.016) | 0.125 |
|  |  |  |  |  |  |  |  |  |  |
| **Model II** |  |  |  |  |  |  |  |  |  |
| **Baseline** |  |  |  |  |  |  |  |  |  |
| **NTUH** |  | 0.001 (0.002) | 0.448 | 0.01 (0.01) | 0.452 |  |  |  |  |
| **CCH** |  | -0.0006 (0.001) | 0.694 | -0.005 (0.01) | 0.704 | -0.001 (0.001) | 0.33 | -0.01 (0.01) | 0.349 |
| **NCKUH** |  | -0.0003 (0.001) | 0.839 | -0.004 (0.01) | 0.731 | -0.002 (0.002) | 0.184 | -0.02 (0.01) | 0.176 |
| **48^th^ week** |  |  |  |  |  |  |  |  |  |
| **NTUH** |  | 0.0003 (0.002) | 0.885 | 0.002 (0.01) | 0.892 |  |  |  |  |
| **CCH** |  | -0.0009 (0.002) | 0.585 | -0.009 (0.01) | 0.528 | -0.0003 (0.001) | 0.817 | -0.003 (0.01) | 0.783 |
| **NCKUH** |  | -0.001 (0.002) | 0.501 | -0.009 (0.01) | 0.481 | -0.001 (0.002) | 0.491 | -0.009 (0.01) | 0.504 |
| **96^th^ week** |  |  |  |  |  |  |  |  |  |
| **NTUH** |  | 0.0008 (0.002) | 0.705 | 0.005 (0.01) | 0.722 |  |  |  |  |
| **CCH** |  | -0.002 (0.002) | 0.265 | -0.015 (0.01) | 0.251 | -0.0005 (0.002) | 0.726 | -0.006 (0.01) | 0.659 |
| **NCKUH** |  | -0.0006 (0.002) | 0.712 | -0.006 (0.01) | 0.691 | -0.001 (0.002) | 0.564 | -0.009 (0.02) | 0.551 |
|  |  |  |  |  |  |  |  |  |  |

Model I: adjusted for age, isoflavone treatment, hospital sites, history of diabetes, hypertension and hyperlipidemia as well as average total mets spent and total calories consumed.

Model II: Model I plus **body mass index**
