# Supplementary material for: A longitudinal analysis of serum adiponectin levels and bone mineral density in postmenopausal women in Taiwan
Source: Sci Rep. 2022 May 16;12:8090. doi: 10.1038/s41598-022-12273-7 (PMC9110357; doi:10.1038/s41598-022-12273-7)
Supplement: Supplementary file 2 — Supplementary Table 2. [file 41598_2022_12273_MOESM2_ESM.docx]

**Supplement Table 2: The association between the changes of serum adiponectin levels and changes of lumbar spine/total proximal**

**femur BMD and t-score using generalized estimating equation (GEE) models by different medical centers**

|  | **Lumbar spine** | | | | **Total proximal femur** | | | | | |
| --- | --- | --- | --- | --- | --- | --- | --- | --- | --- | --- |
| **Serum adiponectin level (mg/dL)** | **BMD** | | **T score** | | **BMD** | | | | **T score** | |
|  | **β (SE)** | **P** | **β (SE)** | **P** | **β (SE)** | | **P** | | **β (SE)** | **P** |
| **Model I** | -0.0004(0.0004) | 0.264 | -0.007(0.003) | 0.031 | -0.001 (0.0004) | | 0.013 | | -0.011(0.003) | 0.001 |
| **NTUH** | -0.0003(0.0008) | 0.754 | -0.001(0.006) | 0.866 | -- | |  | | -- |  |
| **CCH** | -0.0003(0.0005) | 0.526 | -0.002(0.004) | 0.73 | -0.001 (0.0005) | | 0.008 | | -0.011(0.004) | 0.015 |
| **NCKUH** | -0.001(0.0007) | 0.064 | -0.012(0.006) | 0.052 | -0.0003 (0.0006) | | 0.558 | | -0.005(0.005) | 0.321 |
| **Model II** | -0.0004(0.0004) | 0.243 | -0.007(0.003) | 0.027 | -0.001 (0.0004) | | 0.011 | | -0.001(0.003) | 0.001 |
| **NTUH** | -0.0003(0.0008) | 0.743 | -0.001(0.006) | 0.855 | -- | |  | | -- |  |
| **CCH** | -0.0003(0.0005) | 0.526 | -0.002(0.004) | 0.729 | -0.001 (0.0005) | | 0.008 | | -0.011(0.004) | 0.014 |
| **NCKUH** | -0.001(0.0007) | 0.061 | -0.012(0.006) | 0.049 | -0.0003 (0.0006) | | 0.532 | | -0.005(0.005) | 0.301 |
| **Model III** | -0.0006(0.0004) | 0.121 | -0.005(0.003) | 0.143 | -0.001 (0.0004) | | 0.008 | | -0.009(0.003) | 0.008 |
| **NTUH** | -0.0002(0.0008) | 0.811 | -0.0006(0.006) | 0.913 | -- | |  | | -- |  |
| **CCH** | -0.0003(0.0005) | 0.544 | -0.001(0.004) | 0.753 | -0.001 (0.0006) | | 0.009 | | -0.011(0.005) | 0.016 |
| **NCKUH** | -0.001(0.0007) | 0.065 | -0.012(0.006) | 0.053 | -0.0003 (0.0005) | | 0.618 | | -0.004(0.004) | 0.343 |
| **Model IV** | -0.0005(0.0004) | 0.206 | -0.004(0.003) | 0.239 | -0.0008(0.0004) | | 0.026 | | -0.007(0.003) | 0.025 |
| **NTUH** | -0.00005(0.0008) | 0.954 | 0.0004(0.005) | 0.945 | -- | |  | | -- |  |
| **CCH** | -0.0003(0.0005) | 0.598 | -0.001(0.004) | 0.822 | -0.001(0.0005) | | 0.050 | | -0.009(0.004) | 0.029 |
| **NCKUH** | -0.0001(0.0001) | 0.175 | -0.010(0.006) | 0.105 | -0.0001(0.0005) | | 0.838 | | -0.003(0.005) | 0.522 |
|  |  |  |  |  |  | |  | |  |  |
|  | **Lumbar spine** | | | | **Total proximal femur** | | | | | |
| **Serum adiponectin level (mg/dL)** | **BMD** | | **T score** | | **BMD** | | | **T score** | | |
|  | **β (SE)** | **P** | **β (SE)** | **P** | **β (SE)** | **P** | | **β (SE)** | | **P** |
| **Model V** | -0.0005(0.0004) | 0.167 | -0.004(0.003) | 0.195 | -0.0008(0.0004) | 0.029 | | -0.007(0.003) | | 0.028 |
| **NTUH** | -0.0004(0.0008) | 0.606 | -0.002(0.006) | 0.672 | -- |  | | -- | |  |
| **CCH** | -0.0003(0.0005) | 0.578 | -0.001(0.004) | 0.807 | -0.001(0.0005) | 0.013 | | -0.009(0.004) | | 0.026 |
| **NCKUH** | -0.001(0.0007) | 0.154 | -0.009(0.006) | 0.131 | -0.00003(0.0005) | 0.957 | | -0.002(0.004) | | 0.606 |
| **Model VI** | -0.0003(0.0004) | 0.373 | -0.003(0.003) | 0.390 | -0.0007(0.0004) | 0.050 | | -0.006(0.003) | | 0.050 |
| **NTUH** | 0.0003(0.0008) | 0.670 | 0.003(0.005) | 0.568 | -- |  | | -- | |  |
| **CCH** | -0.0003(0.0005) | 0.601 | -0.001(0.004) | 0.820 | -0.001(0.0005) | 0.019 | | -0.009(0.004) | | 0.035 |
| **NCKUH** | -0.0009(0.0007) | 0.196 | -0.009(0.006) | 0.157 | -0.00009(0.0005) | 0.863 | | -0.001(0.004) | | 0.796 |

Model I: adjusted for time and time^2^

Model II: adjusted for age, time and time^2^

Model III: adjusted for age, time, time^2^, isoflavone treatment, hospital sites, history of diabetes, hypertension and hyperlipidemia as well as average total METs spent and total calories consumed.

Model IV: Model III plus body mass index

Model V: Model IV plus bone alkaline phosphatase

Model VI: Model IV plus urinary N-telopeptide of type 1 collagen/creatinitine
